# Niobium-Titanium-Based Photocatalysts: Its Potentials for Free Cyanide Oxidation in Residual Aqueous Effluent

**DOI:** 10.3389/fchem.2020.00099

**Published:** 2020-03-20

**Authors:** Aida Liliana Barbosa López, Isel M. Castro

**Affiliations:** Laboratory of Catalysis Research and New Materials (LICATUC), Department of Chemical, Exact and Natural Science Faculty, University of Cartagena, Cartagena, Colombia

**Keywords:** mining activities, cyanate, ultraviolet irradiation, niobium oxides, Lewis acidity, solid state, titanoniobates

## Abstract

Organic compounds are employed as additives to increase the dissolution speed of gold, in concentrations around 1 g/L when using cyanidation, thereby forming a residual aqueous effluent with high amounts of free cyanides and organic compounds, which generate metallic complexes difficult to degrade. To increase the photodegradation efficiency, promising niobium and titanium porous materials are proposed as photocatalysts, due to their role in simultaneous oxidation and reduction reactions. In the process of cyanide oxidation, NbO_5_ 0.3H_2_O was doped with titanium oxalate (IV) of 0.5, 1, and 1.5%; and HTiNbO_5_ were synthesized, from the mixture of NbO_5_ with TiO_2_ Degussa-P25, by coprecipitation, impregnation, and solid state. The determination of its elemental composition, morphological and textural properties were carried out by using various XRD techniques, Raman spectroscopy, SEM/EDS and acidity by pyridine. The experiments of photocatalytic oxidation of cyanide used one semibatch reactor with ultraviolet irradiation 125 W in a pH range of 9.5–12. The catalyst with the highest percentage of degradation was HTiNbO_5_ 93.7%, which is attributed to the microstructure of the double layer and Lewis acidity sites, followed by NbTi-1% 92.9% and the Nb_2_O_5_.3H_2_O 82.4%, being the majority product cyanate, proposing its mechanism of reaction. Characterization experiments indicated Nb-O-Ti bridges that have been associated with the control of redox properties of the niobium species and Ti-O-Nb = O, which could be generating a greater number of e–H +pairs, increasing the photocatalytic activity. It is considered that the method of synthesis has a strong influence in changing the morphology of the particles such as porosity, specific surface and factors such as the acidity of niobium–based catalysts, which are important to achieving efficiency in degradation. Niobium-Titanium photocatalysts proved to be an excellent new breakthrough in Advanced Oxidation Technologies (AOT), to eliminate cyanide in wastewater from mining activities.

## Introduction

The environmental impact of gold mining in Latin America is being recognized for having implications on public health. In 2013, through the United Nations Organization, 91 countries signed the Minamata agreement, which seeks to protect human health and the environment from the adverse effects of mercury (Pnuma, 2013).

The chemical inputs for extraction are mercury (Hg), sodium cyanide (NaCN), hydrogen peroxide (H_2_O_2_), zinc and activated carbon; however, organic additives based on oil are used for the flotation process of the mineral pine, anthragenic oils or glycine, which pass into leachate waters (Eksteen and Oraby, 2015). Added to this are the formed amalgams that have between 40 and 50 wt % of Hg and between 50 and 60 wt % of other metals such as gold, silver, and copper (Ilyas and Lee, 2018). These residuals interact with cyanide that is usually in excess to form metal complexes, some of a weak nature, Cu ${\left(\text{CN}\right)}_{3}^{-2}$, Zn ${\left(\text{CN}\right)}_{4}^{-2}$, Hg (CN)_2_, Cd ${\left(\text{CN}\right)}_{4}^{-2}$, called “dissociable in weak acids” cyanides, which separate at a pH (4–6), producing environmentally significant concentrations of free cyanide (Elysee, 2009). Other types of Metal-Cyanide complexes are classified as strong, Fe ${\left(\text{CN}\right)}_{6}^{-4}$, Fe ${\left(\text{CN}\right)}_{6}^{-3}$, Au (CN)^−2^, Co ${\left(\text{CN}\right)}_{6}^{-3}$ and require very low pH values pH <2 to dissociate (Dai et al., 2010). The stability of these complexes prevents their removal by physical methods, adsorption, complexation and/or oxidation (Smit and Mudder, 1991).

Advanced Oxidation Processes (AOP) are being researched to improve the removal efficiency of these pollutants associated with cyanidation, which require an affordable source of light (Aguado et al., 2002; Osathaphan et al., 2008; Pineda and Silva, 2010). In return, they own high oxidative power and low selectivity, allowing degradation of compounds of inorganic and organic nature, and their mixtures, including compounds that are not adsorbed in active carbon, are not very volatile and non-biodegradable substances while being in the aqueous phase. In addition, due to their high potential for mineralization, their final waste is less harmful and final disposal is easier; in some cases, it allows the reduction of the metals present (Rivera-Utrilla et al., 2013; Ribeiro et al., 2015). A fundamental part of the success of AOPs lies in the photocatalyst, which must break down a wide range of recalcitrant compounds at room temperature and pressure. Catalytic systems based on niobium (Nb), niobium pentoxide (Nb_2_O_5_), and mixtures of niobium oxide and titanium oxide (Nb_2_O_5_- TiO_2_) are useful for various reactions. Due to the versatility of the Nb-O bond, its redox potential, high acidity, and strong metal support interactions act as an active phase, doping phase and as a support (Ziolek, 2003; Yan et al., 2014; Lopes et al., 2015; Da Silva et al., 2016). It is possible to highlight reactions produced by the photoexcitation of semiconductor compounds that have niobium in their structure. When dispersed in solutions or in mixtures of gases, they promote simultaneous oxide-reduction reactions of the species in the environment, selective oxidations in photooxidation processes of organic compounds and displacement of water. Therefore, they are effective for the generation of molecular hydrogen and reactions that lead to a complete degradation of organic pollutants present in the medium (Linsebigler et al., 1995). Compounds such as hydrated niobium oxide (Nb_2_O_5_ · nH_2_O) and niobium phosphate (NbOPO_4_.2H_2_O) have strong acidic properties on surfaces (Ho = −5.6), corresponding to an acid strength of 70% sulfuric acid (H_2_SO_4_) and solid acid catalysts used.

In particular, the niobic acid known as (Nb_2_O_5_ · *n*H_2_O), which contains large amounts of water, exhibits high catalytic yields for acid catalyzed reactions with the participation or release of water molecules (Carniti et al., 2005). Particular attention is given to niobates, which can be combined with titanium and with divalent metals such as Ca (II), Mg (II), Sr (II), Sn (II), Ba (II), Zn (II), Cd (II), and Pb (II) (Cho et al., 2009; Noh et al., 2012; He et al., 2015; Resende et al., 2019). They show laminar structures, which allow and have optical properties that increase their power (Raba et al., 2015; Caicedo, 2019). The surface of Nb_2_O_5_ species is controlled by hydroxyl chemistry, which is an advantage for photocatalytic reactions where hydroxyl is the active species to carry out oxidation reactions (Nowak and Ziolek, 1999). For the modification of the structures and properties of Nb-based materials, various synthesis methodologies have been used. For example, the Nb_2_O_5_ precipitation using sol gel methods, followed by hydrothermal heating in the presence of surfactants to achieve specific surface increase passing from 78 to 151 m^2^/g, noting that the presence of chloride ions in the surfactant did not cause structural changes in Nb_2_O_5_ (Ayudhya et al., 2008).

Typically, niobium-based photocatalysts can be used in wastewater treatment, discarded in gold mining, due to their complexity, which requires research into new synthesis methods. In the present work, we synthesize new catalytic systems based on titanium and niobium, looking at the effect of the percentage of impregnation on the NbO_5_ · 3H_2_O of the titanium oxalate [Ti (C_2_O_4_)_2_], and comparing the photocatalytic activity of titanoniobiate (HTiNbO_5_) in the degradation of cyanide by tests in a semibatch reactor with ultraviolet irradiation of 125 W. The effects of catalyst concentration, pH and temperature and the importance of metal oxygen species, which can confer good catalytic behavior, were analyzed. The acidic, morphological, and textural properties of the synthesized solids were being examined using XRD, Raman spectroscopy, SEM/EDS, FT-IR, and acidity by pyridine, showing that the synthesized materials titanium niobates represent a great change for cyanide removal requiring further studies using residual effluents extracted from the mines.

## Materials and Methods

### Materials

Two sources of semiconductor oxides were used: TiO_2_ Degussa P-25 (80 anatase and 20% rutile) with a surface area of ≈54 m^2^/g, 0.2031 cm^3^/g and pore size 0.016 A μm, particle size 0.34 μm and Nb_2_O_5_ · 3H_2_O, donated by CBMM (Brazilian Metallurgy Company) 98% purity, whose characteristics include a surface area of 170 m^2^/g before some pre-treatment temperature, pore volume of 0.2 cm^3^/g, and pore size of 2–5 μm (D50). For kinetic experiments, KCN (Panreac) was used as a source of cyanide, HCl (Merck) and NaOH (Carlo Erba) reagent grade to adjust the pH between 9.5 and 12.

### Synthesis of Niobium-Titanium-Based Photocatalysts

#### Synthesis of Titanium Oxalate (Ti (C_2_O_4_)_2_)

The synthesis of Ti (C_2_O_4_)_2_ was performed by the Pechini method, using a rotary evaporator and mechanical agitation (Bernal et al., 1995; Wang et al., 2005; Gallo et al., 2013). The Oxalic Acid (Merck, 98 w%) in excess was diluted in 2-propanol (Merck, 98 w%), in which TiO_2_ (Degussa P-25) was dispersed in a 250 mL reaction vessel. The mixture was permitted to reflux for ~17 h at a constant temperature of 80°C, with a slight bubbling during the reaction time. The white precipitate was dried at 120°C, in an oven at atmospheric pressure for 6 h. The synthesis of NbO_5_ · 3H_2_O (supplied by CBMM) used in this work is reported by Rangel et al. (2019), who explore the sintering of the pyrochloro mineral and subsequent refinement in an electric furnace, where it is defoforized and treated in an alkaline medium for its subsequent calcination. The impregnations of NbO_5_ · 3H_2_O with Titanium oxalate (IV) (0.5, 1, and 1.5 w%) were made wet using Ti (C_2_O_4_)_2_ (0.01, 0.02 mol, and 0.03 mol, respectively), maintaining magnetic stirring at 300 rpm for 3 h. The paste mixture was subjected to ultrasound for 5 min. Subsequently, the solid was dried at 60°C in a vacuum oven for 12 h and calcined at 300°C for 3 h to ensure the best crystallinity of the final compound.

#### Synthesis of Acid Titanoniobiate (HTiNbO_5_) Catalyst

Potassium titanoniobiate (KTiNbO_5_) was taken as a starting material for the synthesis of the precursor of HTiNbO_5_ and was obtained using the methodology described in (Nyman et al., 2003; Saupe et al., 2005; Barbosa and Castro, 2012). For the synthesis of the precursor of KTiNbO_5_, a cation exchange reaction of KTiNbO_5_ was carried out using 4 M hydrochloric acid (HCl) for 3 days, changing the acid solution every 24 h. The KTiNbO_5_ solid produced was washed with deionized water for 5 days, renewing the water every 24 h.

To the remaining solid of the washing, deionized water was added with magnetic stirring at 360 rpm for 1 h and was adjusted to pH at 9, adding dropwise a solution of 40% v/v ammonium hydroxide (NH_4_OH), which was then allowed to stand for 24 h before the supernatant was extracted. The solid formed (precipitate) was siphoned with plenty of water for 5 days, washing the supernatant daily. After that, 2 M H_2_SO_4_ was added, stirring for 2 h and letting it rest for 1 h, washing again for 48 h and drying at 120°C in a vacuum oven for 3 h (Castro and Barbosa, 2012). The materials synthesized for this investigation and their identification are reported in Table 1.

**Table 1:** Synthesized catalytic systems for photocatalytic cyanide removal describing the method used.

**Catalyst**	**Code**	**Preparation method**
Nb_2_O_5_.3H_2_O	NbO	N.A.
titanium oxalate (IV) 0.5%/Nb_2_O_5_ · *3*H_2_O	NbTi-0.5%	Impregnation by wet (Precipitation)
titanium oxalate (IV) 1%/Nb_2_O_5_ · *3*H_2_O	NbTi-1%	Impregnation by wet (Precipitation)
titanium oxalate (IV) 1.5%/Nb_2_O_5_ · *3*H_2_O	NbTi-1.5%	Impregnation by wet (Precipitation)
HTiNbO_5_	HTiNb	Solid state reaction (Thermal fusion)

### Catalytic Reactions

Photocatalytic activity of the synthesized materials is based on niobium that has been evaluated in the cyanide photodegradation reaction using a 0.25 L semi-bach borosilicate reactor for 2 h With a constant stirring at 360 rpm, under irradiation using a low-density UV-C mercury lamp with a wavelength of approximately 254 nm and 125 W, the lamp was inserted vertically in the reactor center, in a borosilicate tube with a thickness of 3 mm, which was submerged inside the cyanide solution. Air was bubbled with a constant flow of 0.6 L/min for 120 min allowing total irradiation of the sample. The system was magnetically agitated at the base of the reactor. The temperature (30°C ± 2) and the pressure were maintained at environmental conditions as it was necessary to work to strongly alkaline conditions, thus the pH was monitored to avoid the formation of volatile HCN. Studies by Augugliaro et al. (1997) establish a range catalyst concentration between 0.05 and 3.0 g/L for cyanide catalytic removal in aqueous phase, with better results when used in high concentrations. For this purpose, we chose a catalyst ratio of 2.85 g/L.

Before irradiation magnetic stirring, the photocatalyst was dispersed in the absence of light for 1 h, until equilibrium was reached. Then, the suspension was irradiated with UV light. The determination and quantification of the concentration of free cyanide was carried out by the potentiometric method following the manual 4500-CN-C standardized methods, using a HANNA Cyanide Ion Selective Electrode HI 4,109 brand cyanide selective ion electrode potentiometer, coupled to a Meter Tabletop pH/mV/°C HI 223.

The solutions used in each of the tests were prepared at a concentration of 100 mg CN-/L, which was standardized. Additionally, the participation of the air and the adsorption of the substrate on the catalyst were determined; the photolysis, with and without air under the experimental conditions was selected to determine the participation in the process of each of these agents. Samples taken from the reaction system at times of 20, 40, 60, 80, 100, and 120 min were centrifuged for 10 min at 4,200 rpm and evaluated in cyanide removal.

The majority product of the free cyanide oxidation that was cyanate was potentiometrically measurement using a pH/mV/°C HI 221 with a HI 4101 HANNA selective ammonium ion electrode, using the standard 4500-CN-L protocol methods for the examination of water and wastewater.

### Characterization of Materials

A Rigaku miniflex II benchtop XRD equipped with (CuK-alpha) λ = 1.5418 A°), operating at 40 Kv and 30 mA of current, has been used to measure the XRD patters exposed surface. The accumulation conditions of scanning were in the range of 5–90° for values (2θ), with a scanning speed of 2°(2θ)/min.

Raman spectroscopic analyses of the phase composition were carried out on a LSI Dimension P1 Raman System spectrophotometer operated with Diode Laser (785 nm), which had a temperature stability of 0.007 nm/°C and was calibrated with Hg/Ar, a data capture effectiveness of 10 ms. The resolution of the spectrum was 1.5 cm^−1^. Textural and morphological properties were studied using a JEOL JSM-840 scanning electron microscope to achieve photomicrographs with magnifications of 2000 x and 3000 x at 20 kV, coupled with an EDS electron emission unit. For a more detailed observation of the layered structure of the titanoniobiate, a Cryo-SEM Zeiss DSM 960 equipment with a LaB6 cathode was used, operated at 20 KV, with graphite as conductive material. For the purpose of improving signal, contrast and coverage were covered with a layer of fine gold (BIO-RAD Semiconductor Sputter Coater E5175). Initially, the sample was cooled at the maximum possible speed using nitrogen snow.

The nature of the acidic sites on the surface of the solids prepared has been determined by FTIR of adsorbed pyridine using the same equipment with a special accessory, such as the infrared cell.

The infrared spectra were taken at room temperature, using a Shimadzu FTIR-8400S (120 V) FT-IR spectrometer, in absorbance mode in a spectral range of 4.000 cm^−1^-400 cm^−1^, with a resolution of 2 cm^−1^. The catalytic precursors were pulverized taking between 30 and 50 mg and pressing them with ~38 metric tons of pressure, which was deposited in the special infrared cell in stainless steel, with CaF_2_ windows cooled with water, to form a 1 cm thick tablet.

The samples were first pretreated *in situ* at 200°C with air for 2 h and a flow of 100 mL/min, allowed to cool to 25°C, taking a spectrum to verify the cleanliness of the surface. Following this, the gases were evacuated over 2 h by passing a smooth flow of helium and exposing in a pyridine flow (Air Liquide, 98.8%, vapor pressure 3.3kPa) for 1 h at 200°C, and another experiment at 300°C, recording the FT-IR spectrum at room temperature. Next, the physiadsorbide pyridine on the catalyst was removed by heating with air at 100°C for 1 h.

## Results and Discussion

### Catalysts Characterization

**Figure 1:**
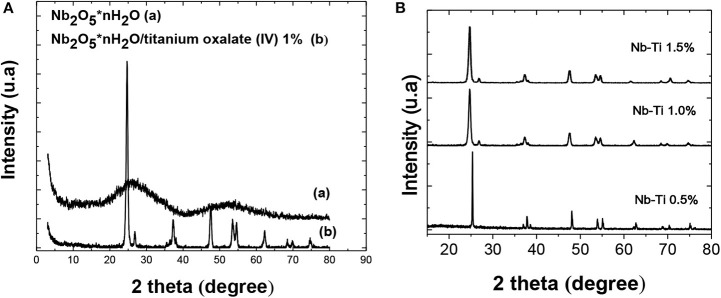
XRD pattern for **(A)** Nb_2_O_5_ · *n*H_2_O (JCPDS-ICDD file 19-862), (a) Nb_2_O_5_ · *n*H_2_O/titanium oxalate (IV) 1%, (b)-XRD patterns for Nb_2_O_5_ · *n*H_2_O catalysts impregnated with titanium oxalate with different loads **(B)** 0.5%, 1% and 1.5% JCPDS-ICDD file 30-872; JCPDS-ICDD file 27-1312.

The XRD analysis shows that the Nb_2_O_5_ · nH_2_O has an amorphous nature. Wide signals are distinguished with maximums in (2 Θ) 10, 24, and 52° (Figure 1), and the phases have been identified using the Powder Diffraction File (PDF) database (JCPDS, International Center for Diffraction Date). The broad bands could be related to the presence of distortions in the NbO_5_ groups due to the broad radius of the Nb^+5^, which obstructs the coupling with the anionic oxygen of the tetrahedron (Védrine et al., 1996; Ruiz et al., 2004; Ayudhya et al., 2008). The coordination of hydration waters present in the Nb_2_O_5_ generates a more amorphous structure.

**Figure 2:**
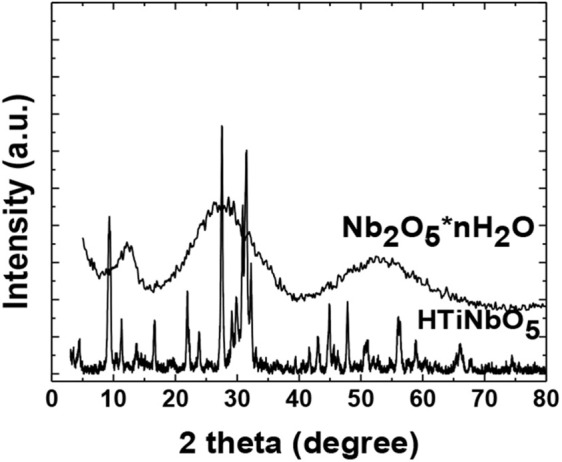
XRD pattern of Nb_2_O_5_-n H_2_O (JCPDS-ICDD file 19-862), and HTiNbO_5_ (JCPDS 72-1076), (JCPDS 71-1747), (JCPDS No. 21-1272).

The DRX pattern for catalysts synthesized with titanium oxalate under wet impregnation for a short interval of (2Θ) showed a significant increase in crystallinity compared to the precursor Nb_2_O_5_ · nH_2_O (Figure 2), corresponding to a compound with different morphology and crystallinity, due to a re-arrangement of the niobium polyhedral units in the dissolution process and a subsequent recrystallization. This is in accord with some authors, who attribute the occurrence of this to hydrothermal synthesis (Murayama et al., 2014). The most intense XRD signal centered at 2Θ = 24.0 and 28.2° could be related to agglomerated nanospherical particles produced in NbO_5_, formed in the presence of oxalate, conforming to JCPDS-ICDD file 27-1312 (do Prado and Oliveira, 2017). It has been reported that a chelated polyhedral type can be formed with niobium through the oxygen atoms of oxalate. Preventing the formation of skewed chains, this helps to control the recrystallization processes, acting as a template and favoring the nanorods formation (Truong et al., 2012).

It could be seen that the peak's intensity, corresponding to the anatase phase of TiO_2_ (2Θ = 63.15°), changes with the percentage of titanium oxalate (IV), favoring its formation at low impregnation loads of 0.5 and 1% (Onfroy et al., 2007). In addition, there are some characteristic peaks of titanium oxalate (IV) at 27,62, 36,54, 48,43°, which coincide with the diffractogram reported by Dambournet et al. (2011), as well as characteristic peaks of the crystalline phases of TiO_2_ Degussa P-25 anatase and rutile at 63,15 and 69,97°, respectively (Souzanchi et al., 2013).

The X-ray diffraction pattern for the titanium niobiate acid catalyst (HTiNbO_5_), synthesized under a solid state reaction (thermal fusion), showed an increase in crystallinity against the Nb_2_O_5_ precursor (Figure 2). As is frequently the case with this method of synthesis, the diffractogram showed a mixture of crystalline phases Nb_2_O_5_, TiO_2_ and HNbTiO_5_, correlated with more than four very intense reflections observed in the diffractogram. The presence of interlaminar structures of HTiNbO_5_ is appreciated in a cluster of three reflections observed in the XRD diffractogram, centered at 28.5 and 31.7° and the peak at 10.62°, with different interplanar space measurements that could not be calculated, but that could indicate different morphologies associated to the present phases. The presence of nanorods was observed by the 23.6° centered signal identified according to JCPDS-ICDD file 30-872. The reflection peaks appreciated in the diffractogram centered at 22.48 and 28.21° could be associated with spherical agglomerated particles belonging to niobium oxides that can be seen in JCPDS-ICDD file 27-1312. On the other hand, reflection peaks were also observed at 24.14 and 48.15°, corresponding to the anatase active phase of TiO_2_. Using the Scherrer equation, crystallite sizes of Nb_2_O_5_ were found for poor impregnations (NbTi-0.5%) (Table 2). Sizes of 6.61nm corresponding to isolated nanocrystals were obtained for loads greater than NbTi-1.0 and NbTi-1.5%, the crystallite size increased to 19.07 and 19.18 nm, respectively. These nanocrystal sizes show great potential for catalytic reactions.

**Table 2:** Particle size using the Scherrer equation.

**Sample**	**Θ**	**β**	**Dp (nm)**
Nb_2_O_5_ · *n*H_2_O	27.6	0.1530	0.94
	52.9	0.2070	1.16
Nb_2_O_5_ · *n*H_2_O/ titanium oxalate (IV) 0,5%	25.1	0.0243	6.61
	37.3	0.0112	16.40
Nb_2_O_5_ · *n*H_2_O/ titanium oxalate (IV) 1%	24.7	0.0084	19.07
	37.3	0.0112	16.39
Nb_2_O_5_ · *n*H_2_O/ titanium oxalate (IV) 1,5%	24.7	0.0000	19.18
	37.3	0.0110	16.39
HTiNbO_5_	10.3	0.0007	20.32
	28.5	0.0005	34.77

The morphology of the catalysts was evaluated by scanning electron microscopy (SEM).

**Figure 3:**
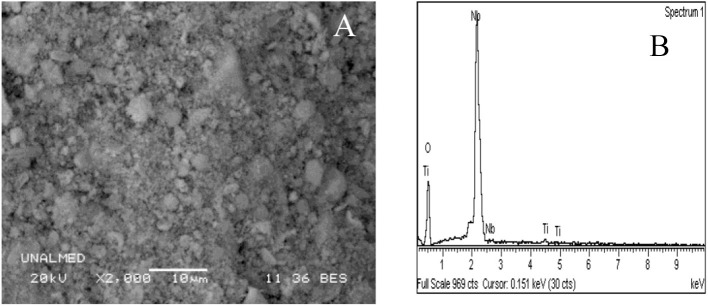
Scanning microscopy images for catalyst **(A)** NbTi-1%, and **(B)** EDS analysis of NbTi-1% particles.

**Figure 4:**
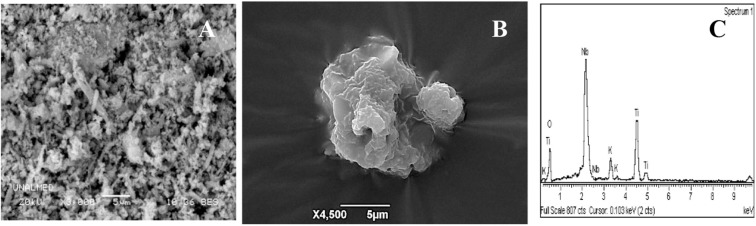
Scanning microscopy images for catalyst **(A)** HNbTiO_5_ 3000X, 20kV, **(B)** pore detail 4500X, and **(C)** EDS analysis of HNbTiO_5_ particles.

For Nb_2_O_5_ solids obtained using oxalate as an NbTi template with loads of 0.5, 1, and 1.5% (Figure 3), very heterogeneous amorphous conglomerates of spherical particles with different crystallite sizes are shown, as well as very small spherical crystals associated with Nb_2_O_5_ as reported in the XRD data discussion. EDS analyses show variations in the concentrations of titanium, niobium, and oxygen of the solid. The SEM photomicrograph and SEM-EDS analysis for the HTiNbO_5_ catalyst, with an increase of 3.000 X (Figure 4), shows a morphological transformation with respect to the NbTi, as a mixture of several phases can be seen.

**Figure 5:**
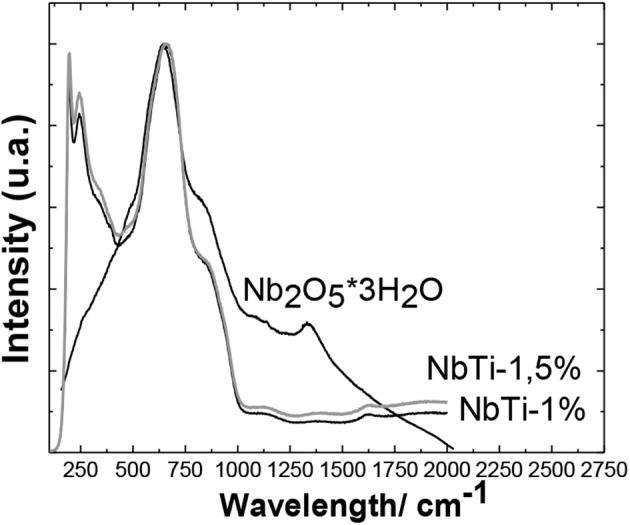
Raman spectrum of Nb_2_O_5_ · 3H_2_O; Nb_2_O_5_ · *n*H_2_O catalysts impregnated with titanium oxalate 1 and 1.5%.

Cane/parallelepipeds shapes appear (Kruk and Jaroniec, 2001), as do large structures in randomly organized layer shapes that report belonging to NbO (Cai et al., 2012), which generate anisotropic growth in layers and sheets. Quasi-spherical granular aggregates that form links related to TiO_2_ have been observed. The EDS microprobe shows different concentrations of titanium in several positions on the surface—an expected result as due to the presence of a mixture of crystalline phases, the cation exchange did not give 100%, leaving an atomic percentage of 2.9% of potassium (K) on the surface. The atomic percentage for niobium was 39.1% and that for titanium was 20.2%, which was consistent with the proportions used to perform the synthesis of this catalyst. The HTiNbO_5_ catalyst indicates the presence of porous structures of different sizes (Figure 4B). This particular morphology could contribute to catalytic activity, favoring intraparticle pores that would facilitate the diffusion of cyanide molecules in the cyanate oxidation process. Raman spectroscopy of Nb_2_O_5_ *n*H_2_O (Figure 5) showed a band around 500–700 cm^−1^ due to Nb-O stretches and a poorly resolved shoulder around 290 cm^−1^, which was assigned to the bond weak Nb-OH (Barros et al., 2008).

**Figure 6:**
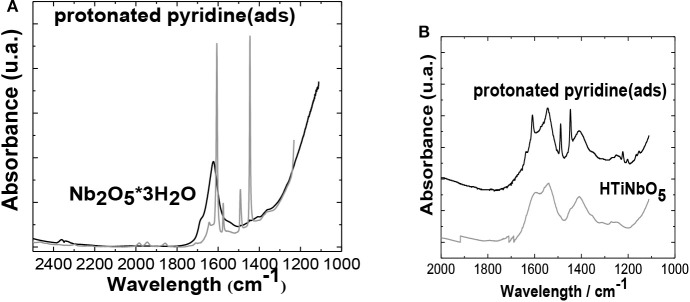
Infrared spectrum obtained after adsorption of pyridine for the catalysts. **(A)** NbTi-1% and NbTi-1.5%, **(B)** HTiNbO_5_.

The NbO sample contains slightly distorted polyhedra, identified by a band that comes out at around 1,350 cm^−1^. When titanium is added to the NbO sample, we observe that this characteristic band of the polyhedra in the Raman spectrum of the NbTi- (0.5, 1, and 1.5%) disappears. This is consistent with the results of the X-ray diffraction, which demonstrate the ways in which crystallinity improved with the adding of the Ti to the crystal network of the NbO cell. The strong and wide band of around 650 cm^−1^ is also assigned to symmetrical stretches of the Nb-O link polyhedra. The shoulder around 730 cm^−1^ appears due to the Nb-O-Nb link flexion mode (Burcham et al., 1999; Schulte et al., 2007). These bands have also been assigned to link Nb-O-Nb and symmetrical stretches Nb-O of Nb_2_O_5_ or symmetrical stretches Nb = O on the surface, respectively (Kominami and Oki, 2001; Nakamoto, 2009). The Raman spectrum of the Nb_2_O_5_ *n*H_2_O samples doped with titanium oxalate (IV) are shown in Figure 5. The sample of Nb_2_O_5_ *n*H_2_O/titanium oxalate (IV) 0.5% is not presented as there was no significant difference between the Raman spectrum of this sample and the Raman spectrum of Nb_2_O_5_ *n*H_2_O without doping. In the Raman spectra of Nb_2_O_5_ *n*H_2_O/titanium oxalate (IV), a very defined band appears at 100 cm^−1^, related to the Nb = O species in highly distorted octahedral NbO_6_ (Ayudhya et al., 2008). Additional bands below 500 cm^−1^, which are not present in the Raman spectrum of Nb_2_O_5_ *n*H_2_O, such as the band in 390 cm^−1^, were assigned to Ti-O vibrational stretching of the Ti-O-Ti dimeros (Dutta et al., 2000). At 200 cm^−1^, a band representative of the anatase phase of TiO_2_ Degussa P-25 can be found, and finally at 234 cm^−1^ a band assigned to metal-O vibration modes appears. Around 1,600 cm^−1^, a band appears, which is assigned to C-O stretches (Stockenhuber et al., 1993). Pyridine adsorption, followed by infrared spectroscopy, was used for the identification of acid sites in the synthesized catalysts (Figure 6). After subjecting the NbO, NbTi-1% and HTiNb0_5_ to the adsorption of pyridine, bands corresponding to the vibrational mode of the species Piridinium ion (PyrH +, Bronsted sites) appeared, which were obtained at around 1,545 and 1,638 cm^−1^. The coordinated linkage of pyridine with Lewis acid sites (Pyr-L, lewis sites) was observed at around 1,445 and 1,610 cm^−1^. Physisorbed pyridine or hydrogen bonds of pyridine showed characteristic signals around 1,440 and 1,600 cm^−1^. The band around 1,489 cm^−1^ was common to all catalysts, corresponding to the vibrations of the protonated PyrH + ion and coordinated bonds of the Pyridine (Pyr-coord) (Bassan et al., 2013). This indicates that the NbO has both types of acid sites (Lewis and Bronsted), though predominantly Lewis-type acid sites, NbTi-1% has predominantly Bronsted-type acidity (Chatterjee and Dasgupta, 2005), while HTiNbO_5_ only has Lewis type acid sites.

### Photocatalytic Reactions

To analyze the influence of each of the parameters involved in the catalytic process, the reaction targets were made with solutions prepared in the laboratory, with an initial concentration of 100 mg/L CN-. It can be observed that the concentration of catalyst cyanide and air and the photolysis did not exceed 7% removal of free cyanide, while the photocatalytic process showed the fastest and most complete degradation.

**Figure 7:**
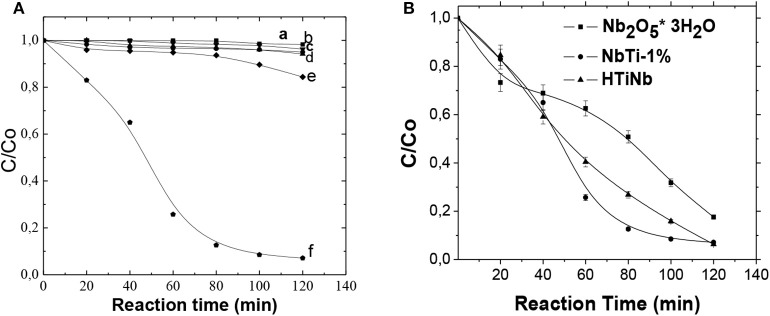
**(A)** Evaluation of reaction targets in the semibatch reactor, (a)-■-CN- + air, (b)-•-CN- + ligh, (c)-▴-CN- + catalyst + air, (d)-▾- CN- + catalyst, (e)-♦- CN- + light + air, (f)-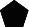
- CN- + light + air + catalyst. **(B)** Degradation of CN- using NbO, NbTi and HTiNb as catalysts, [catalyst]: 2.85g/L; pH: 9.5; t: 120 min.

In order to increase photocatalytic efficiency in the cyanide oxidation process, niobic acid (Nb_2_O_5_ · 3H_2_O) was impregnated with titanium oxalate (IV) in different proportions (NbTi-0.5%; NbTi-1%; NbTi-1.5%), to achieve enhancement of electron capture and generate greater numbers of *OH radicals, or develop peroxo surface groups due to their strong oxidizing properties. In addition, the (HTiNb), from the mixing of NbO with TiO_2_ Degussa-P25 was synthetized for evaluation them in the photocatalitic removal of free cyanide for 2h in aqueous medium, by virtue of also being a semiconductor material. The niobic acid (Nb_2_O_5_ · 3H_2_O) was impregnated with titaniumoxalate (IV) with different loads (NbTi-0.5%; NbTi-1%; NbTi-1.5%). The behavior of the degradation curves can be seen in Figure 7. The best results were observed from the HTiNbO_5_ catalyst with 93.7%, very close to NbTi-1%, which reached a yield of 92.9%. The Nb_2_O_5_ reached only 82.4%. The results of cyanide photodegradant activity for NbO catalysts, NbTi-1, and HTiNb% are shown in Table 3.

**Table 3:** Influence of the catalyst composition on the reduction of free cyanide.

**Time (min)**	**NbO (%)**	**NbTi- 1% (%)**	**HTiNb (%)**
0	0	0	0
20	26.7	17.0	15.4
40	31.1	29.0	40.9
60	37.4	74.3	59.6
80	41.2	87.4	73.2
100	48.2	91.5	84.2
120	82.4	92.9	93.7

The redox activity of these niobium systems could be related to bridge oxygen that is generated between Nb-O-Ti (Domen et al., 1996). The addition of titanium to the NbO surface in different proportions (NbTi-0.5, NbTi-1, NbTi-1.5%) favored varied crystalline shape and intrinsic defects in these semiconductor oxides.

Developing ability to play their role as intermediate energy states, promoting a decrease or inhibition in the speed of recombination e–h+, obtaining increased catalytic activity. On the other hand, the incorporation of TiO_2_ in the crystalline network of NbO, if we assume the tetravalence of titanium, there would be a pair of valence electrons for the Nb cation being generated a new electronic density of occupied states, in the valence band region. This additional number of electrons in the valence band could form a non-binding type Nb-derivative, while Nb located in the binding state would persist on the surface, where electrons could be more easily excited and lead to the formation of e-pairs (e–h+), which are responsible for the photocatalytic activity on the surface of the catalysts.

In the case of HTiNbO_5_ that registered a better result in the degradation of cyanide, this was possibly due to its laminar structure and redox properties in this catalyst being more active in the oxidation of cyanate, as well as that its Lewis acidity favored a main product, which was cyanate (Wachs et al., 1996).

Looking at the structure of the interlayers of HTiNbO_5_ with very thin laminates, one might think that the transfer of load carriers would be more efficient in this type of structure as the e-h pair would migrate to the catalyst surface more quickly and reactionary with adsorbed species. Thus, recombination of it would be minimized, making these catalysts more efficient in cyanide removal. Thin lamellaes in the titanoniobiate with a larger area would act as electron collectors and have a high charge mobility. This interaction would allow the titanium electrons present in P-25 to be transferred to the niobiate, increasing the time of recombination of the electron pair—hollow, improving its stability and activity.

The metals in the catalyst have two possible oxidation states—for titanium Ti^+3^ and Ti^+4^ and for niobium Nb^+4^ and Nb^+5^, varying the number of vacancies, which are given by interstitial oxygen (Liu et al., 2010). If it is assumed that the Nb is in the +5 state and the Ti in a +4 state, a stoichiometric reduction of Ti^+4^ to Ti^+3^ is given for each niobium available in the network, forming a more reactive species ${\text{Nb}}_{\text{Ti}}^{*}$ represented by the reaction according to (Ruiz et al., 2004) (1):

(1) $$\begin{array}{l} \frac{1}{2}Nb_{2}O_{5}+\;Ti_{Ti}^{x}\;\to Nb_{Ti}^{\cdot }+\;e^{-}+h^{+}+TiO_{2}+\frac{5}{4}O_{2} \end{array}$$

For the synthesized photocatalyst that has niobites groups in its structure, the interlaminar hydrogen bonding of the O-H···O type would promote better electronic conduction, favoring the oxidation process. On the other hand, the OH groups that contain the interlamines in the vertices of the octahedra with terminal groups Nb=O and Ti = O bonds appreciated by Raman can act synergistically, increasing the stability of the solid. The Ti-O-Nb type bonds appreciated by Raman spectroscopy would help preserve the active phase against the photocatalytic reaction, favoring the removal of free cyanide (Shintaro and Ugur, 2006). The acid sites that possess these high crystallinity laminar structures of the Ti^4+^(OH)Nb^5+^ type found by adsorption-desorption of pyridine, confer a wide variety of ion exchange, in which the hydrogen atom present in the interlamines could be displaced and anchor the contaminant to carry out the free cyanide oxidation process (Takagaki et al., 2004). By XRD analysis, it was observed that the TiO_2_ phase present in HTiNbO_5_ was anatase, which is active for the oxidation of free cyanide to cyanate, which could follow the mechanism described below:

(2) $$\begin{array}{l} TiO_{2}+hv\to TiO_{2}\left(2h^{+}+2e^{-}\right) \end{array}$$

(3) $$\begin{array}{l} {\frac{1}{2}O}_{2}+2e^{-}+H_{2}O\to 2OH^{-} \end{array}$$

(4) $$\begin{array}{l} 2OH^{-}+2h^{+}\to 2OH \end{array}$$

(5) $$\begin{array}{l} CN^{-}+2OH\to OCN^{-}+H_{2}O \end{array}$$

(6) $$\begin{array}{l} {2OCN}^{-}+O_{2}\to {2CO}_{2}+N_{2} \end{array}$$

Resulting in the general equation according to Harraz and Abdel-Salam (2013)

(7) $$\begin{array}{l} {2CN}^{-}+{2O}_{2}\;\vec{TiO_{2}{/H}_{2}O/hv}\;{2CO}_{2}+N_{2} \end{array}$$

### Evaluation of Reaction Products, Cyanate Determination

**Figure 8:**
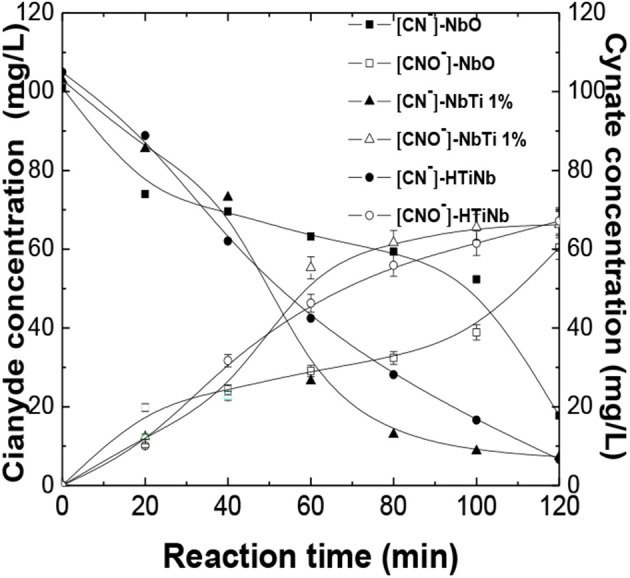
Formation of cyanate as the main product of the oxidation of free cyanide in aqueous medium exposed to UV radiation.

The cyanate concentration was monitored as the main reaction product of cyanide photocatalytic oxidation reaction, performed indirectly by measuring the concentration of ammonium. Figure 8 demonstrates a comparison of the decrease in cyanide concentration in parallel to the formation of cyanate in a semibatch type reactor. It was appreciated that cyanide oxidation indirectly occurred through hydroxyl radicals for the catalysts used in this study. The cyanate formation rate sequence was HTiNbO_5_ >NbTi-1%>NbO, which may be due to an effect synergistic between niobium and titanium for the generation of the e–h+ pairs, which at the same time produce the HO· radicals. Due to the reaction between the HO- adsorbed and the h+, which in the case of titanium intervenes with the ≡ TiO· species and in the case of niobium it has not been identified which species acts, but being in the aqueous environment results in the formation of HO· (Chatterjee and Dasgupta, 2005). Moreover, the different types of acidity developed in the catalysts influenced the catalytic performance. For the solid with better performance, HTiNbO_5_, we believe that an excess of positive charge given by the niobium led to the preferential development of Lewis sites. These surface sites are capable of receiving electrons and show a greater affinity for the electrons donor nitrogen's that exists in cyanide (Corma and Garcia, 2002), facilitating the transfer of oxygen to the molecule to form cyanate.

## Conclusions

Through modifications in the method of obtaining catalysts based on Nb_2_O_5_, it was possible to improve its crystallinity and obtain different morphologies from rods, semi-spherical aggregates, and sheets, in addition to differences in porosity and varied particle sizes, which gave preferential acidity Lewis, as was the case of HTiNbO_5_. This factor influenced its effectiveness in oxidation reactions of free cyanide. The best catalytic behavior was the HTiNbO_5_ with a removal of 93.7% in 2 h, using a catalyst concentration of 2.85 g/L, pH: 9.5 to 100 ppm concentration of the cyanide solution. With these results, the photocatalytic technology option is favored in the treatment of toxic compounds with a low cost, using UV-Vis light and air in a time of 120 min at moderate temperature and pressure conditions. We were able to obtain catalysts with surface acidity characteristics higher than TiO_2_, which were more active in the cyanide oxidation reaction. We went from a catalyst with Lewis and Bronsted acidity such as low-performance catalytic niobic acid, to a solid Bronsted acidity such as titanium oxalate (IV)/Nb_2_O_5_-3H_2_O, which increases the oxidation of the photolith intended to be destroyed. The enrichment of Lewis-type acidity led to an increase in the rate of cyanide degradation for titanoniobiate acid catalyst. Considering that TiO_2_ is of a basic nature, it is clear that the addition of niobium improved its acidity and activity.

## Data Availability Statement

All datasets generated for this study are included in the article/supplementary material.

## Author Contributions

Conceptualization, validation, formal analysis, resources, writing-original draft preparation, supervision, project management, and acquisition of funds: AB. Methodology, software, research, and visualization: AB and IC. Data handling: IC. Writing: AB and IC.

### Conflict of Interest

The authors declare that the research was conducted in the absence of any commercial or financial relationships that could be construed as a potential conflict of interest.
